# The evaluation of prognostic value of serum tumor marker in ovarian tumors

**DOI:** 10.1186/1753-6561-9-S1-A56

**Published:** 2015-01-14

**Authors:** S Sharief, S Riaz

**Affiliations:** 1 Rak Medical and Health Sciences University, Ras Al Khaimah, United Arab Emirates

## Background

Ovarian cancer still remains the deadliest cancer of the female reproductive tract. Unfortunately, most cases are diagnosed in the late stages of the disease. The most useful tumor marker in the detection of ovarian cancer is cancer antigen (CA) 125. The greatest problem of CA 125 determination is its lack of specificity. Aim To evaluate the clinicopathological features of ovarian tumors and their correlation with the serum markers like CA125 in detecting ovarian tumors.

## Methods

A retrospective analysis of ovarian tumor patients, treated at Gynecology and Obstetric department in Saqr hospital between 2011-2013, was performed. All the socioepidemiological and clinicopathological features were retrieved from the patient’s files.

## Results

Number of Cases: 45 All the 45 patients included in the study had benign ovarian masses. Age Range: 15-64 yrs, type of ovarian tumor percentage (%), Dermoid cyst (teratoma), 35.5 serous tumors, 33.1 Chocolate cyst, 13 Paraovarian cyst, 13 Follicular cysts, 2.2 Malignancy, 2.2 Approximately 35.5% of the cases were mature cystic teratoma, followed by serous cystadenoma (33.1%). The right ovary was involved in 56.2%, the left ovary in 31.2% and bilateral ovaries were involved in 12.5%. The serum levels of CA125: The mean serum level was 90.5±99.5 (range 7.0-294).

## Conclusions

The diagnostic value of serum CA125 in distinguishing a benign from a malignant ovarian mass is important, but the main limit of serum CA125 is that it may be high in benign diseases, especially in the reproductive age.

